# Acute Effects of Electronic Cigarette Inhalation on the Vasculature and the Conducting Airways

**DOI:** 10.1007/s12012-019-09516-x

**Published:** 2019-04-08

**Authors:** Lukasz Antoniewicz, Amelie Brynedal, Linnea Hedman, Magnus Lundbäck, Jenny A. Bosson

**Affiliations:** 1 Division of Internal Medicine, Department of Clinical Sciences, Karolinska Institutet, Danderyd University Hospital, 182 88 Stockholm, Sweden; 2 Department of Public Health and Clinical Medicine, Umeå University, Umeå, Sweden; 3 Department of Public Health and Clinical Medicine, Occupational and Environmental Medicine, The OLIN Unit, Umeå University, Umeå, Sweden; 4 Division of Nursing, Department of Health Science, Luleå University of Technology, Luleå, Sweden; 5 Division of Cardiovascular Medicine, Department of Clinical Sciences, Karolinska Institutet, Danderyd University Hospital, Stockholm, Sweden

**Keywords:** Electronic cigarettes, ENDS, Arterial stiffness, Pulse wave velocity, Augmentation index, IOS, e-Cig

## Abstract

The use of electronic cigarettes has increased exponentially since its introduction onto the global market in 2006. However, short- and long-term health effects remain largely unknown due to the novelty of this product. The present study examines the acute effects of e-cigarette aerosol inhalation, with and without nicotine, on vascular and pulmonary function in healthy volunteers. Seventeen healthy subjects inhaled electronic cigarette aerosol with and without nicotine on two separate occasions in a double-blinded crossover fashion. Blood pressure, heart rate, and arterial stiffness measured by pulse wave velocity and pulse wave analysis were assessed at baseline, and then at 0 h, 2 h, and 4 h following exposure. Dynamic spirometry and impulse oscillometry were measured following vascular assessments at these time points, as well as at 6 h following exposure. e-Cigarette aerosol with nicotine caused a significant increase in heart rate and arterial stiffness. Furthermore, e-cigarette aerosol-containing nicotine caused a sudden increase in flow resistance as measured by impulse oscillometry, indicating obstruction of the conducting airways. Both aerosols caused an increase in blood pressure. The present study indicates that inhaled e-cigarette aerosol with nicotine has an acute impact on vascular and pulmonary function. Thus, chronic usage may lead to long-term adverse health effects. Further investigation is warranted.

**Electronic supplementary material:**

The online version of this article (10.1007/s12012-019-09516-x) contains supplementary material, which is available to authorized users.

## Introduction

Tobacco smoke has long been associated with damage and disease in nearly every organ of the body, with the most common being various types of cancer as well as cardiovascular and respiratory disease [1]. Due to an increased public awareness of these adverse health effects in addition to stricter laws and regulations, the Western world has witnessed a steady decline in cigarette smoking over the last few decades [2]. On the other hand, the electronic cigarettes (e-cigarettes), introduced on the market in 2006, have gained heavily in popularity on a global scale [3].

All e-cigarette systems are comprised of a battery, a cartridge/tank with liquid (e-liquid), and an atomizer which contains a wick, coil, and heating element. The wick draws the e-liquid into the coil and when activated the e-liquid is heated, the aerosol is then inhaled by the e-cigarette user. The e-liquid is based on a mixture of vegetable glycerin and propylene glycol and may contain added flavorings and/or varying amounts of nicotine [4]. Both are common food additives and also found in numerous industrial, commercial, and pharmaceutical products. Propylene glycol is a known eye and airway irritant and commonly used in fog machines [5]. However, it is important to note that it is still unclear whether either has negative health effects when heated and inhaled.

To date, there are a handful of human experimental studies examining the health effects of e-cigarette usage. Two studies have demonstrated lower amounts of urinary biomarkers for oxidative stress and carcinogens in chronic e-cigarette users compared to smokers [6, 7]. Another study showed impaired flow mediated dilation and an increase in serum biomarkers for oxidative stress following exposure to electronic cigarette aerosol (ECA) with nicotine [8]. Our group has recently demonstrated that ten puffs of ECA with nicotine mobilized endothelial progenitor cells in healthy volunteers [9]. We speculated that this was due to endothelial activation or damage. However, none of these studies could differentiate if the observed effects were due to the nicotine content or other contributing factors found in the ECA. Moheimani et al. observed an increase in cardiac sympathetic nerve activity in healthy volunteers exposed to ECA with nicotine but not following ECA without nicotine or sham smoking [10]. Two further studies found that inhalation of ECA with nicotine but not without nicotine caused an increased arterial stiffness at 5–10 min following inhalation, a known independent risk factor for both myocardial infarction and stroke [11–13].

Vardavas et al. demonstrated that exposure to ECA increased airway obstruction measured by impulse oscillometry (IOS), but not by conventional spirometry [14]. IOS is commonly used clinically in the pediatric population to assess obstructive pulmonary diseases. This method is highly reproducible and it allows a deeper understanding of small airway diseases and may even diagnose pre-clinical obstructive states [15]. Another study showed that passive ECA exposure increased fractional exhaled nitric oxide (FeNO), an airway inflammation marker commonly assessed in asthmatics [16]. However, other studies have pointed towards a decrease in FeNO upon ECA inhalation [14, 17].

To further understand acute vascular and pulmonary effects of ECA and nicotine, we performed a study in which healthy volunteers were exposed to active e-cigarette inhalation with or without nicotine.

## Methods

### Study Design and Subjects

Employing a randomized, double-blinded, crossover design, 17 healthy occasional users of tobacco products (max ten cigarettes/month), inhaled 30 puffs of ECA with or without nicotine during a 30-min period on two separate occasions. The wash out period was a minimum of 1 week. Prior to the study days, volunteers had to abstain from alcohol and caffeine for 12 h, from heavy exercise for 24 h and from other tobacco and nicotine-containing products for 14 days. All subjects underwent a preliminary clinical examination including ECG, dynamic spirometry, pregnancy test, and routine blood tests including full blood count, electrolytes, creatinine, apolipoproteins, HbA1c, aPTT, and PT. Exclusion criteria included any form of cardiovascular, respiratory, systemic or chronic disease, symptoms of infection or inflammation within 2 weeks prior to study start, BMI ≥ 30 or pregnancy.

### e-Cigarette Exposure

The e-liquid base consisted primarily of 49.4% propylene glycol, 44.4% vegetable glycerin, and 5% ethanol without any added flavorings (Valeo laboratories GmbH, Germany). Premixed e-liquids with and without added nicotine were used (19 mg/ml and 0 mg/ml resp.). A variable mod third-generation e-cigarette was used (eVic-VT, Shenzhen Joyetech Co., Ltd., China). The same settings were used for all exposures (temperature 230 °C, effect 32 W, resistance 0.20 Ω). A dual coil nickel atomizer was used. All exposures were performed in a well-ventilated, temperature-controlled room. Volunteers inhaled 30 puffs from the e-cigarette for 30 min, with each puff lasting approximately three seconds.

### Measurements

**Fig. 1:**
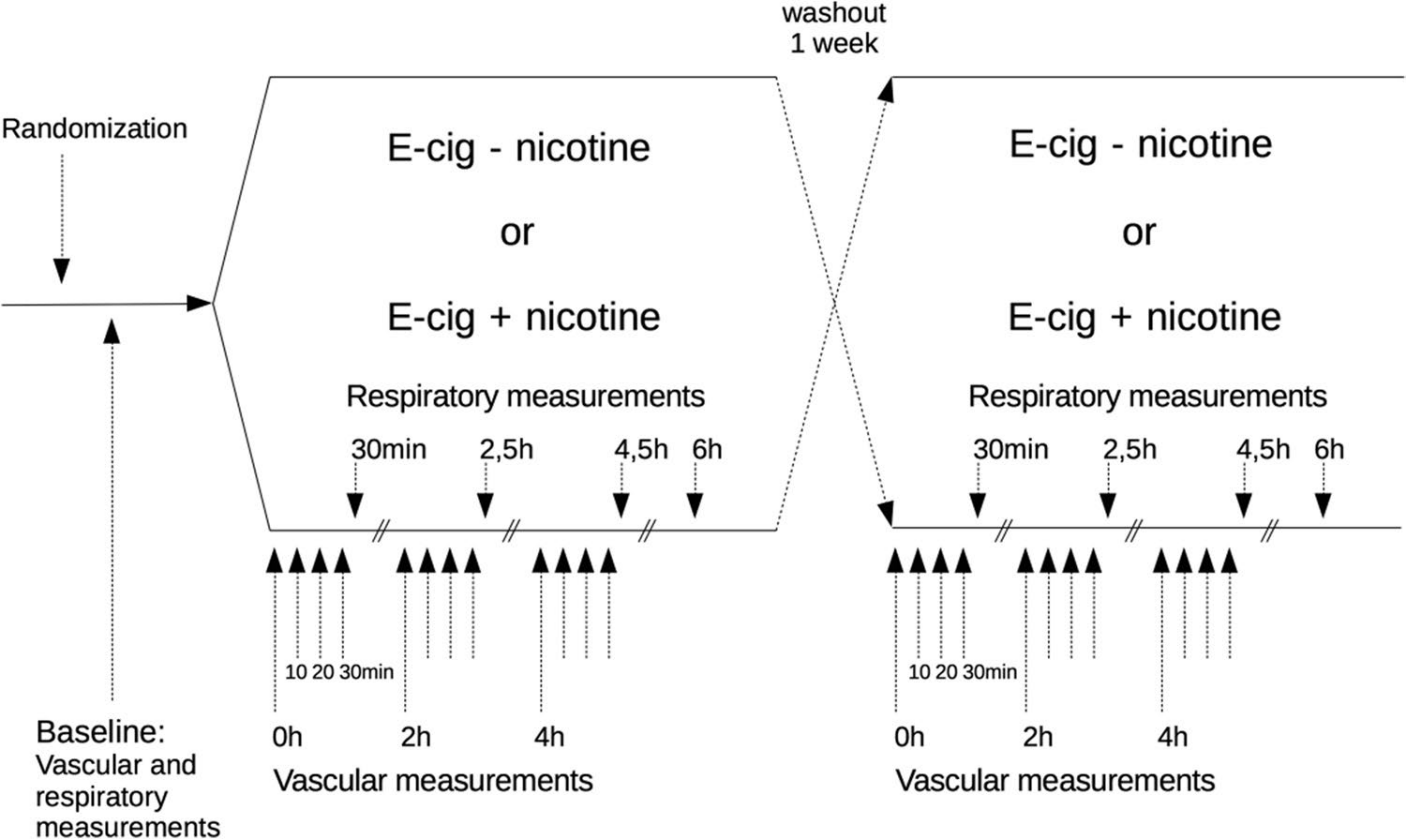
Study design

Vascular measurements included heart rate (HR), systolic and diastolic blood pressure (SBP, DBP), and arterial stiffness. Pulmonary measurements consisted of dynamic spirometry, impulse oscillometry (IOS), and fractional exhaled nitric oxide (FeNO). Vascular measurements were performed at baseline and then every 10 min for 30 min following the inhalations at 0 h (directly following exposure), 2 h, and 4 h (Fig. 1). Pulmonary measurements were carried out following vascular measurements as well as at 6-h post-exposure.

Using a double-blinded, crossover study design, volunteers were randomized to e-cigarette inhalation either with or without nicotine as their first exposure. Vascular measurements included systolic and diastolic blood pressure, heart rate, and arterial stiffness and were measured at baseline and following exposure, immediately (0 h), 2 h, and 4 h afterwards. These measurements were performed in 10-min intervals over 30 min. Respiratory measurements included dynamic spirometry, impulse oscillometry (IOS), and fractional exhaled nitric oxide (FeNO) and were performed directly following the vascular measurements and additionally at 6-h post-exposure.

#### Vascular Measurements

All assessments were performed in a quiet, temperature-controlled room by one investigator with volunteers in a semi-supine position. Blood pressure and heart rate were measured using a validated semi-automatic oscillometric sphygmomanometer (Omron M7, Omron Healthcare Europe B.V., Hoofddorp, NL).

Arterial stiffness was assessed using pulse wave analysis and pulse wave velocity (PWV). PWV was determined by the Vicorder™ system (Skidmore Medical, Bristol, UK). This system measures the pulse transit time between two inflatable cuffs; one placed around the neck and the other around the thigh in order to register the pulse waves in the carotid and femoral arteries. Pulse wave analysis was assessed by micromanometer applanation tonometry (Millar Instruments, Texas, USA) on the right radial artery and analyzed with SphygmoCor™ software (AtCor Medical, Sydney, Australia), which then evaluates the aortic pulse pressure waveform via a validated mathematical transfer function. This waveform comprises a forward pressure wave, originating from the ventricular contraction of the heart and a reflected wave caused by the peripheral vascular resistance. Augmentation index and augmentation pressure were calculated from this waveform. Since augmentation index is inversely proportional to HR it was normalized for a HR at 75 bpm (AIx75). All measurements complied within the SphygmoCor™ quality control criteria [18].

#### Respiratory Measurements

All respiratory measurements were performed with volunteers sitting in an upright position using a standard nose clip.

FeNO was assessed with the Niox Mino system (Aerocrine AB, Solna, Sweden) in accordance with instructions from the manufacturer. Dynamic spirometry was assessed using a Jaeger Masterscope spirometer (Beckton Dickinson and Co., New Jersey, US), following European Respiratory Society (ERS) and American Thoracic Society (ATS) guidelines [19]. Three consecutive measurements of technically acceptable quality were collected and the highest value reported.

IOS was evaluated with a tremoFlo™ device (Thorasys Inc., Montreal, Canada). All measurements were performed according to the manufacturer’s guidelines and following ERS standards [15]. Pulmonary resistance (R) and reactance (X) were measured at oscillation frequencies from 5 to 37 Hz. Low frequencies (5 Hz) penetrate deep into the lung periphery, whereas mid-frequencies (19 Hz) merely reach the upper airways. Hence, resistance at 5 Hz (R5 Hz) reflects the whole respiratory system, whereas resistance at higher frequencies (R19 Hz) reflect the upper airways. Furthermore, the difference between low and high frequencies is used to specify the resistance in the peripheral airways (R5–19 Hz). Reactance at 5 Hz (X5 Hz) illustrates the elastic properties of the lung and obstruction of the smaller airways. Resonant frequency (fres) is referred to as the point where reactance is zero and the area under the curve from X5 Hz to fres is referred to as the area of reactance (AX). Three measurements with good technical quality were reported as mean values.

### Cotinine Analysis

Serum cotinine levels at baseline were measured using a commercially available ELISA method (Calbiotech, Spring valley, CA, US) in accordance with the manufacturer’s instructions.

### Statistical Analysis

The statistical analyses were performed with SPSS 24.0 (IBM Corporation, NY, US) and GraphPad Prism 7.0 (GraphPad Software Inc., CA, US). Prior to analysis, data were checked for normality both visually and by Shapiro–Wilk test. Skewed variables (FeNO, R5–19, AX, fres) were checked for outliers and one subject had to be removed from FeNO analysis due to overall high values during both exposures. Skewed variables were analyzed following logarithmic transformation and two-way repeated measures ANOVA was performed. If Mauchly’s test for sphericity was violated, Greenhouse–Geisser corrected results were presented. Within-subject contrasts were analyzed to compare baseline values to all other time points. *P*-values of < 0.05 were considered statistically significant. Statistical analyses were performed by blinded investigators.

Power analysis calculations based on our previous particulate matter exposure study results, which also employed these methods, determined a sample size of *n* = 15 [20].

The study was approved by the local Ethics Review Board in Umeå. The study was performed in accordance to the Declaration of Helsinki and with the written informed consent of all participants.

## Results

Two subjects were excluded due to elevated cotinine values at baseline, indicating non-compliance with the study protocol. Fifteen subjects (nine females, six males, mean age 26 ± 3 years), all healthy, sporadic smokers, were included into the analysis. Routine blood samples as well as BMI and waist circumference were taken prior to the study. Mean values of the subject characteristics are shown in Online Resource 1.

### Vascular Measurements

**Fig. 2:**
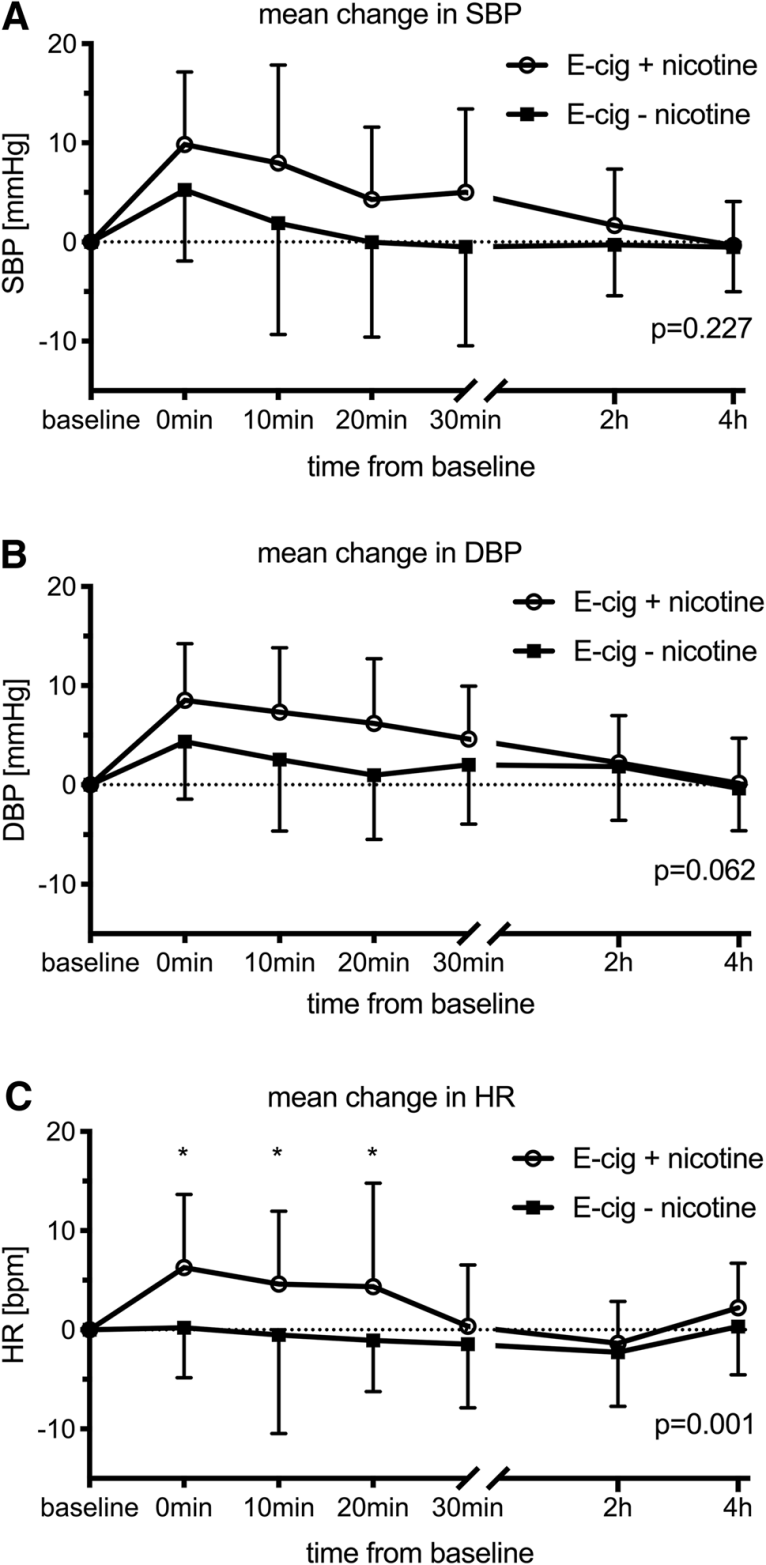
Effects on blood pressure and heart rate. Mean change in vascular measurements with standard deviations from baseline following exposure to e-cigarette aerosol with and without nicotine. **a** systolic and **b** diastolic blood pressure (SBP, DBP), **c** heart rate (HR). *P*-values are presented for multiple measures ANOVA for the interaction variable of ‘time × exposure.’ *Denotes significant change from baseline due to exposure (contrast for ‘time × exposure’)

**Fig. 3:**
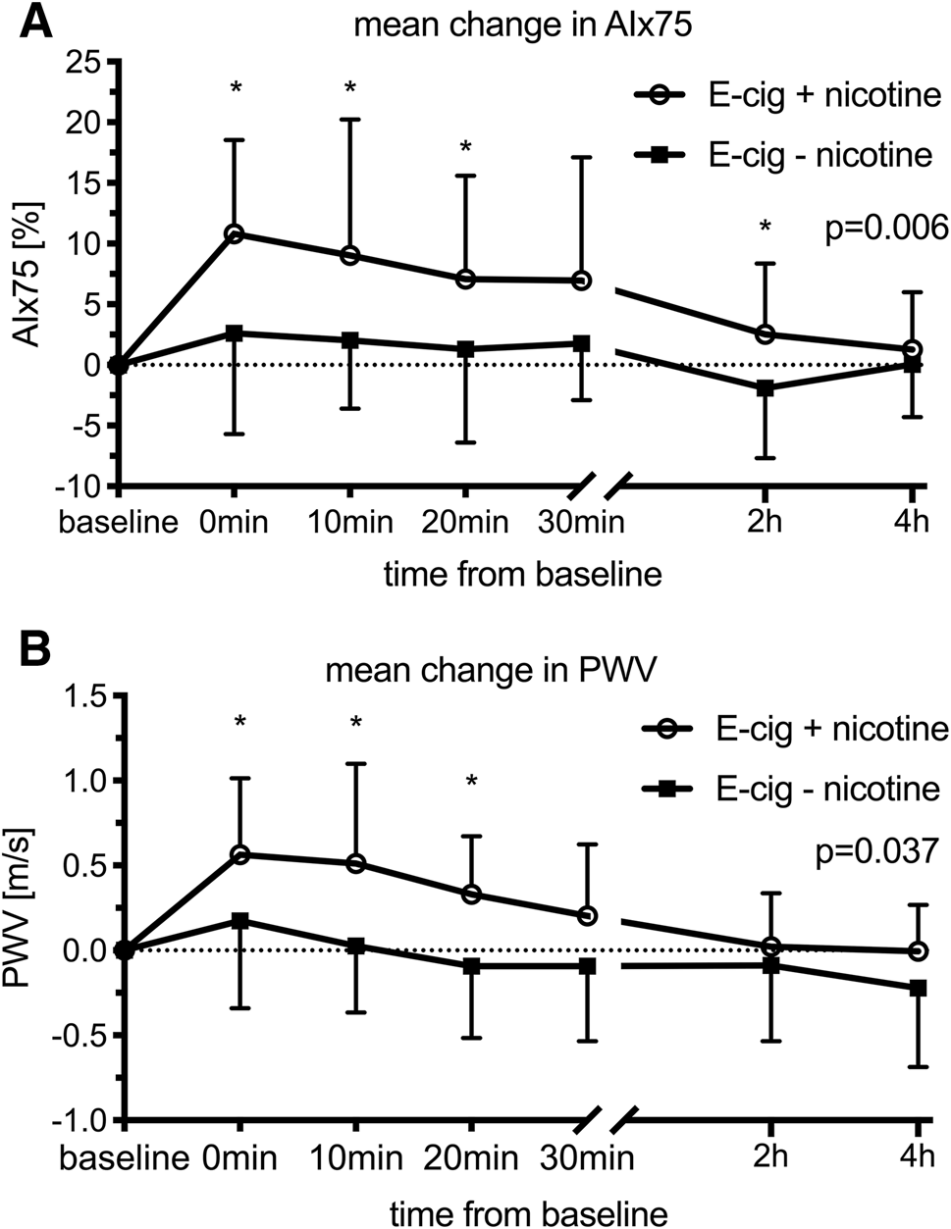
Effects on arterial stiffness. Mean change in arterial stiffness with standard deviations from baseline following exposure to e-cigarette aerosol with and without nicotine. **a** heart-rate corrected augmentation index (AIx75) and **b** pulse wave velocity (PWV). *P*-values are presented for multiple measures ANOVA for the interaction variable of ‘time × exposure.’ *Denotes significant change from baseline due to exposure (contrast for ‘time × exposure’)

All vascular measurements are shown in Table 1. Following both exposures (with and without nicotine), there was a significant increase in SBP and DBP that remained elevated for 10 and 30 min, respectively (Fig. 2, Table 1). HR, PWV, and AIx75 increased significantly following exposure to ECA with nicotine and remained elevated for 20 min as compared to ECA without nicotine (Figs. 2, 3, Table 1).

**Table 1:** Vascular measurements at baseline and following exposure to electronic cigarette aerosol (ECA) with and without nicotine

	ECA	Baseline	Post exposure						ANOVA, *P*-values	
			0 min	10 min	20 min	30 min	2 h	4 h	Time	Time × exposure
SBP	+ nicotine	109.4 ± 9.5	119.3 ± 9.5^†^	117.4 ± 13^†^	113.7 ± 10.3	114.5 ± 12	111.1 ± 10.1	109.1 ± 9.5	< 0.001	0.227
	− nicotine	109.3 ± 10.3	114.5 ± 13.2^†^	111.2 ± 16.1^†^	109.3 ± 15.5	108.8 ± 15.4	109 ± 10.2	108.8 ± 11.7		
DBP	+ nicotine	70.3 ± 5.7	78.9 ± 5.9^†^	77.7 ± 6.6^†^	76.5 ± 6.6^†^	74.9 ± 5.8^†^	72.6 ± 5.4	70.5 ± 6.6	< 0.001	0.062
	− nicotine	70.2 ± 5.8	74.5 ± 6.9^†^	72.7 ± 8.2^†^	71.1 ± 8.1^†^	72.2 ± 8^†^	72 ± 6.5	69.8 ± 6.6		
HR	+ nicotine	65.4 ± 8.5	71.7 ± 11.3*	70 ± 12.4*	69.7 ± 12.9*	65.7 ± 10.7	64 ± 9.9	67.6 ± 10.9	0.015	0.001
	− nicotine	63.8 ± 9.7	64 ± 10.7	63.3 ± 12.2	62.7 ± 8.4	62.3 ± 9.2	61.5 ± 9.4	64.1 ± 9.9		
PWV	+ nicotine	5.8 ± 0.8	6.4 ± 0.8*	6.3 ± 0.9*	6.1 ± 0.9*	6 ± 0.8	5.8 ± 0.8	5.8 ± 0.9	< 0.001	0.037
	− nicotine	6.2 ± 0.9	6.4 ± 1	6.2 ± 0.9	6.1 ± 0.8	6.1 ± 0.9	6.1 ± 0.8	6 ± 0.8		
AIx75	+ nicotine	− 5.1 ± 9.5	5.7 ± 11*	3.9 ± 13.2*	2 ± 11.1*	1.9 ± 10.1	− 2.6 ± 11*	− 3.8 ± 10.4	< 0.001	0.006
	− nicotine	− 2 ± 9.2	0.6 ± 12.8	0 ± 10.7	− 0.7 ± 12.9	− 0.3 ± 10.7	− 3.9 ± 10.7	− 2 ± 9.5		

*P*-values are presented for multiple measures ANOVA for ‘time’ and the interaction variable of ‘time × exposure’

*SBP* systolic blood pressure, *DBP* diastolic blood pressure, *HR* heart rate, *PWV* pulse wave velocity, *AIx75* heart-rate corrected augmentation index

*Denotes significant change from baseline due to exposure (contrast for ‘time × exposure’)

^†^Denotes significant change from baseline, not influenced by exposure (contrast for ‘time’)

### Respiratory Measurements

**Fig. 4:**
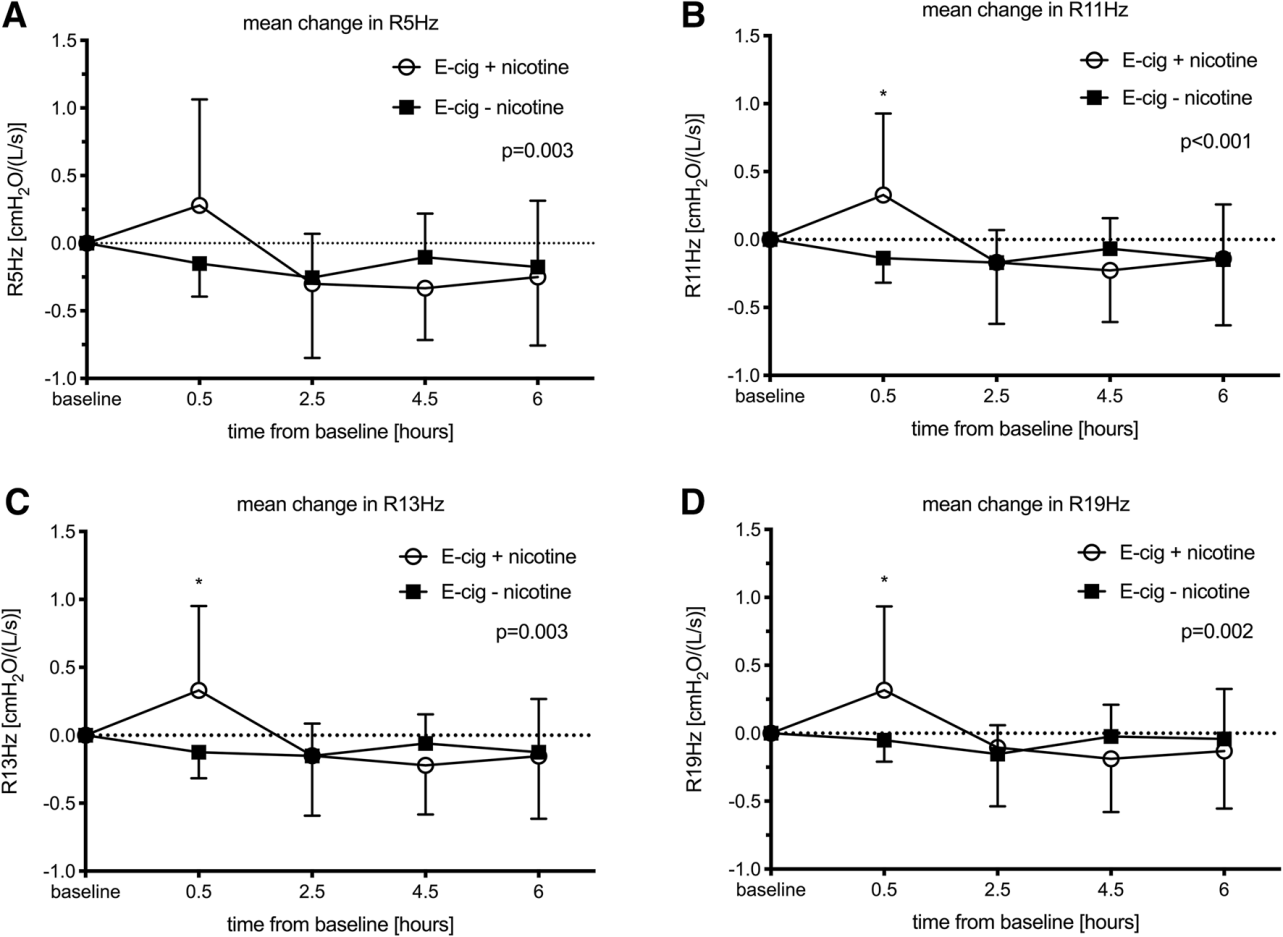
Effects on airways measured by impulse oscillometry (IOS). Mean change and standard deviations from baseline in flow resistance at 5, 11, 13, and 19 Hz (R5 Hz, R11 Hz, R13 Hz, R19 Hz). *P*-values are presented for multiple measures ANOVA for the interaction variable of ‘time × exposure.’ *Denotes significant change from baseline due to exposure (contrast for ‘time × exposure’)

Results from respiratory measurements are presented in Table 2. Thirty minutes following exposure to ECA with nicotine, flow resistance at 5, 11, 13, 17, and 19 Hz was significantly increased (Fig. 4, Table 2). Resonance frequency (fres) decreased at 6 h following inhalation of ECA without nicotine. Flow reactance at 5 Hz (X5 Hz), the difference of R5 Hz and R19 Hz (R5–19 Hz), and reactance area (AX) remained unaffected following both inhalation exposures (Fig. 4, Table 2).

**Table 2:** Respiratory measurements at baseline and following exposure to electronic cigarette aerosol (ECA) with and without nicotine

	ECA	Baseline	Post exposure				ANOVA *P*-values	
			0.5 h	2 h	4 h	6 h	Time	Time × exposure
R5 Hz	+ nicotine	3.57 ± 0.73	3.85 ± 0.93	3.27 ± 0.88	3.24 ± 0.66	3.32 ± 0.80	0.001	0.003
	− nicotine	3.41 ± 0.75	3.26 ± 0.70	3.15 ± 0.64	3.30 ± 0.73	3.23 ± 0.72		
R11 Hz	+ nicotine	3.19 ± 0.55	3.52 ± 0.74*	3.02 ± 0.72	2.96 ± 0.54	3.05 ± 0.67	0.002	< 0.001
	− nicotine	3.09 ± 0.67	2.95 ± 0.61	2.92 ± 0.51	3.02 ± 0.65	2.95 ± 0.63		
R13 Hz	+ nicotine	3.18 ± 0.55	3.51 ± 0.77*	3.03 ± 0.70	2.96 ± 0.53	3.03 ± 0.64	0.002	0.003
	− nicotine	3.07 ± 0.67	2.94 ± 0.60	2.92 ± 0.53	3.01 ± 0.65	2.94 ± 0.64		
R17 Hz	+ nicotine	3.18 ± 0.55	3.48 ± 0.75*	3.03 ± 0.66	2.96 ± 0.53	3.03 ± 0.61	0.002	0.010
	− nicotine	3.05 ± 0.68	2.97 ± 0.61	2.91 ± 0.57	3.00 ± 0.69	2.95 ± 0.65		
R19 Hz	+ nicotine	3.23 ± 0.55	3.55 ± 0.74*	3.13 ± 0.67	3.04 ± 0.56	3.10 ± 0.61	0.004	0.002
	− nicotine	3.09 ± 0.69	3.04 ± 0.64	2.94 ± 0.58	3.06 ± 0.71	3.05 ± 0.68		
X5 Hz	+ nicotine	− 0.91 ± 0.29	− 0.85 ± 0.28	− 0.83 ± 0.31	− 0.81 ± 0.30	− 0.82 ± 0.35	0.057	0.980
	− nicotine	− 0.92 ± 0.32	− 0.85 ± 0.30	− 0.81 ± 0.33	− 0.82 ± 0.34	− 0.81 ± 0.28		
R5− 19 Hz	+ nicotine	0.34 ± 0.42	0.30 ± 0.43	0.14 ± 0.34	0.20 ± 0.49	0.22 ± 0.35	0.058	0.314
	− nicotine	0.32 ± 0.41	0.22 ± 0.29	0.22 ± 0.37	0.24 ± 0.47	0.18 ± 0.26		
AX	+ nicotine	3.48 ± 2.41	3.27 ± 2.15	2.70 ± 2.19	2.87 ± 2.56	3.02 ± 2.40	0.155	0.281
	− nicotine	3.64 ± 2.64	3.03 ± 1.67	2.90 ± 1.89	4.27 ± 3.85	2.57 ± 1.37		
fres	+ nicotine	12.28 ± 3.97	12.06 ± 3.18	10.86 ± 2.57	11.20 ± 3.19	11.73 ± 3.36	0.018	0.042
	− nicotine	12.44 ± 3.66	11.70 ± 2.70	11.54 ± 2.99	11.92 ± 3.35	11.06 ± 2.19*		
VC	+ nicotine	5.01 ± 1.23	4.92 ± 1.18^†^	4.94 ± 1.22^†^	4.96 ± 1.18	4.96 ± 1.19	0.020	0.636
	− nicotine	5.02 ± 1.21	4.98 ± 1.21^†^	4.96 ± 1.20^†^	5.00 ± 1.20	4.97 ± 1.20		
FEV1	+ nicotine	3.82 ± 0.76	3.84 ± 0.79	3.86 ± 0.82	3.85 ± 0.81	3.87 ± 0.80	0.096	0.788
	− nicotine	3.86 ± 0.76	3.86 ± 0.78	3.90 ± 0.77	3.90 ± 0.77	3.89 ± 0.80		
FeNO	+ nicotine	12.36 ± 2.87	12.00 ± 3.55	13.91 ± 3.21^†^	13.09 ± 3.36	11.36 ± 2.98	0.022	0.067
	− nicotine	11.82 ± 3.87	12.91 ± 4.04	12.91 ± 4.01^†^	12.18 ± 3.25	11.27 ± 3.77		

Impulse oscillometry: Flow resistance at 5, 11, 13, 17, and 19 Hz (R5 Hz, R11 Hz, R13 Hz, R17 Hz, R19 Hz), reactance at 5 Hz (X5 Hz), the difference of R5 Hz and R19 Hz (R5–19 Hz), reactance area (AX) and resonance frequency (fres). Dynamic spirometry: Vital capacity (VC) and forced expiratory volume in one second (FEV_1_). Fractional exhaled nitric oxide (FeNO). *P*-values are presented for multiple measures ANOVA for ‘time’ and the interaction variable of ‘time × exposure’

*Denotes significant change from baseline due to exposure (contrast for ‘time × exposure’)

^†^Denotes significant change from baseline, not influenced by exposure (contrast for ‘time’)

FeNO increased significantly at 2 h after both exposures (ECA with and without nicotine). Vital capacity (VC) decreased following exposure to ECA with and without nicotine and remained decreased after 2 h. FEV_1_ did not change significantly over time.

## Discussion

To the best of our knowledge, this is the first comprehensive study in human volunteers that examines acute vascular as well as respiratory effects of electronic cigarette aerosol (ECA) inhalation, both with and without nicotine.

This study shows an acute increase in arterial stiffness, both in terms of PWV and AIx75, following exposure to ECA with nicotine, with a return to baseline values 30-min post-exposure. Increased arterial stiffness is a blood pressure-independent risk factor for cardiovascular events such as myocardial infarctions and stroke [21]. Recently, two other studies demonstrated that a short exposure to ECA with nicotine caused increased arterial stiffness directly following exposure, however, the duration of this change was not assessed in these studies [11, 12]. Applying a Mobil-O-Graph, one pilot study indicated that the increase in arterial stiffness following ECA with nicotine occurs during the first 20 min following exposure and then returns to baseline values afterwards [22]. The present study confirms these findings with two well-established methods for the measurement of arterial stiffness.

There is an ongoing debate on which role nicotine plays in the pathophysiology of atherosclerosis and whether if, and how it may accelerate vascular disease [23]. There are many pathways through which nicotine may cause negative effects on the cardiovascular system. Nicotine has been demonstrated to elicit strong sympathomimetic effects, diminish coronary blood flow, impair endothelial function, enhance inflammation and arteriogenesis, as well as cause insulin resistance [23]. Epidemiological data that correspond with these findings are sparse, as most people tend to use tobacco products as opposed to products containing solely nicotine. Investigating oral snus usage may to some extent reflect long-term nicotine effects, as this product contains high amounts of nicotine and smaller amounts of known health hazardous compounds associated with combustible tobacco [24]. Snus use has been demonstrated to be associated with increased mortality following myocardial infarctions and stroke as well as increased risk of type II diabetes [25–28]. However, transdermal nicotine replacement therapy seems to be safe in patients with cardiovascular disease [29]. It is not unlikely that the pathophysiological effect of nicotine is determined by pharmacokinetics; nicotine administered in tablet form does not give the very quick and high rise in blood nicotine levels as seen following cigarette smoking, inhalation of ECA, or oral snus use [30–32].

In the current study, impulse oscillometry exhibited conducting airway obstruction directly following exposure to ECA-containing nicotine. In 2013, Vardavas et al. demonstrated that a short exposure to ECA caused a rapid increase in airway obstruction [14]. Our study shows a similar impact on the conducting airways, yet only following inhalation of nicotine-containing ECA. These results correspond with findings that inhaled nicotine alone has airway obstructing features in a dose-dependent manner [33]. Itsaso Garcia-Arcos et al. demonstrated that mice exposed to ECA-containing nicotine displayed an increased cytokine expression as well as airway hyperreactivity, in addition to lung tissue destruction normally associated with COPD [34]. This indicates that inhaled nicotine may have additional adverse pulmonary effects compared to the already known systemic effects when administered orally.

It is well known that smoking cessation leads to dramatically improved lung function and reduced airway inflammation, notably so in asthmatic patients. This was demonstrated by Chaudhuri and colleagues, who studied asthmatic patients that quit smoking compared to asthmatic patients who continued to smoke conventional cigarettes [35]. After only 6 weeks of smoking cessation, FEV_1_ had increased with 407 ml compared to those who continued smoking. In a small retrospective study, Polosa et al. investigated asthmatic patients who quit smoking conventional cigarettes and switched to e-cigarette use and demonstrated a comparatively smaller increase in FEV_1_ of around 100 ml following a full 12 months of e-cigarette use [36]. Furthermore, a recently published epidemiological study highlighted e-cigarette use as a risk factor for asthma as well as more severe asthmatic episodes among high school students [37]. These findings, combined with our novel data that added nicotine in ECA likewise causes airway obstruction, point towards e-cigarettes as a poor choice for aiding in smoking cessation, particularly for individuals with bronchial hyperreactivity.

We observed a small yet significant decrease in vital capacity (VC) and a marginal significant increase in FeNO following both exposures. Schober et al. showed a similar increase in FeNO consequent to second-hand e-cigarette exposure, whereas other studies have reported no effect or even reduced levels of FeNO after e-cigarette usage [14, 16, 17]. Changes in VC have not been observed in any other previous studies [14, 17]. These observations are difficult to fully interpret as the changes were minor and lie within repeatability range. Therefore, further studies are needed to clarify whether these findings have any significant clinical impacts following short- or long-term e-cigarette usage [19].

### Study Limitations

IOS, spirometry, and FeNO measurements did not start directly following ECA inhalation. They were performed after the vascular assessments, i.e., 30 min after exposure. Our study protocol is based on the impact the exertion of respiratory measurements may have on the vascular assessments as well as our findings in previous particle exposure studies where vascular effects have been demonstrated at earlier time points than the respiratory effects [20]. Therefore, we cannot exclude a possible impact of ECA on pulmonary measurements during the initial 30 min.

All study participants were young occasional smokers (maximum of ten cigarettes per month). Even though the cumulative cigarette exposure was quite low, we cannot fully exclude that smoking may have affected baseline values of our measurements.

## Conclusions

This study systematically investigates the acute vascular and respiratory effects of e-cigarette aerosol, with and without added nicotine, in healthy volunteers, employing an array of well-validated, non-invasive methods. Our findings suggest that the increase in arterial stiffness and conducting airway obstruction seen following ECA inhalation is primarily caused by the added nicotine in ECA and may translate to clinical repercussions, particularly in susceptible populations as well as with chronic use.

## Electronic supplementary material

Below is the link to the electronic supplementary material.

Supplementary material 1 (DOCX 14 kb)
