# Supplementary material for: Acute Effects of Electronic Cigarette Inhalation on the Vasculature and the Conducting Airways
Source: Cardiovasc Toxicol. 2019 Apr 8;19(5):441–50. doi: 10.1007/s12012-019-09516-x (PMC6746878; doi:10.1007/s12012-019-09516-x)
Supplement: Supplementary file 1 — Supplementary material 1 (DOCX 14 kb) [file 12012_2019_9516_MOESM1_ESM.docx]

|  | mean | SD |
| --- | --- | --- |
| age [years] | 26 | 3 |
| BMI [kg/m^2^] | 24.4 | 3.0 |
| waist [cm] | 82.2 | 8.0 |
| Hb [g/L] | 136 | 14 |
| WBC [x 10^9^/L] | 6.2 | 1.8 |
| platelets [x 10^9^/L] | 280 | 53 |
| Na [mmol/L] | 141 | 1 |
| K [mmol/L] | 4.0 | 0.2 |
| creatinine [µmol/L] | 73 | 14 |
| HBA1C [mmol/mol] | 35 | 3 |
| ApoA [g/L] | 1.54 | 0.28 |
| ApoB [g/L] | 0.83 | 0.19 |
| ApoB/ApoA ratio | 0.55 | 0.16 |
| PT/INR | 1.0 | 0.1 |
| aPPT [s] | 29.4 | 1.7 |

**Online Resource 1.** Mean values with standard deviations (SD) of routine blood samples as well as age, BMI and waist circumference at inclusion.
